# Social Working Memory: Neurocognitive Networks and Directions for Future Research

**DOI:** 10.3389/fpsyg.2012.00571

**Published:** 2012-12-21

**Authors:** Meghan L. Meyer, Matthew D. Lieberman

**Affiliations:** ^1^Psychology Department, University of CaliforniaLos Angeles, CA, USA

**Keywords:** mentalizing, working memory, default mode network, social neuroscience, social cognitive affective neuroscience

## Abstract

Navigating the social world requires the ability to maintain and manipulate information about people’s beliefs, traits, and mental states. We characterize this capacity as *social working memory* (SWM). To date, very little research has explored this phenomenon, in part because of the assumption that general working memory systems would support working memory for social information. Various lines of research, however, suggest that social cognitive processing relies on a neurocognitive network (i.e., the “mentalizing network”) that is functionally distinct from, and considered antagonistic with, the canonical working memory network. Here, we review evidence suggesting that demanding social cognition requires SWM and that both the mentalizing and canonical working memory neurocognitive networks support SWM. The neural data run counter to the common finding of parametric decreases in mentalizing regions as a function of working memory demand and suggest that the mentalizing network can support demanding cognition, when it is demanding social cognition. Implications for individual differences in social cognition and pathologies of social cognition are discussed.

Whether keeping track of friends’ perspectives during conversation, a roomful of colleagues’ beliefs during a conference, or the political ideology of someone we just met, we constantly juggle social cognitive information. Smooth social interaction requires keeping track of various amounts of social information, such as the particular characteristics and relationships among people. Indeed, the “social brain hypothesis” suggests that the fundamental evolutionary constraint leading to increased human brain size, relative to body size, was the need to manage social cognitive demands (Dunbar, 1998).

In cognitive psychology, the process commonly associated with holding multiple pieces of information in mind simultaneously is known as working memory. While there is a great deal of research on the brain mechanisms guiding working memory, tests of working memory have almost exclusively focused on cognitive or perceptual information and have not examined *social working memory (SWM)* for the kinds of social cognitive information that is important for successful social interaction. There are at least two critical barriers that likely prevented research on SWM in the past. First, past research finds similar patterns of behavioral performance across social and non-social cognitive processing demands (Kinderman et al., 1998; German and Hehman, 2006; Apperly et al., 2007). It has therefore been taken for granted that both forms of information processing rely on one working memory system. However, brain imaging research suggests that social cognitive and non-social cognitive information processing rely on distinct brain systems (Kampe et al., 2003; Fox et al., 2005; Mitchell et al., 2005; Ciaramidaro et al., 2007), suggesting that SWM and cognitive working memory (CWM) may rely on distinct, though perhaps correlated, neural mechanisms. Second, the dominant paradigms in social neuroscience show little-to-no variability in the amount of information they require, or working memory demand (Fletcher et al., 1995; Brunet et al., 2000; Walter et al., 2004). The past decade of research has focused on which brain regions engage in a binary fashion to social relative to non-social cognitive tasks. While this has been useful in delineating brain networks engaged in social cognition, it has overshadowed the possibility that these systems are sensitive to SWM demands and show variability in activation across individuals.

We recently reported findings suggesting that SWM may rely on both social cognition and canonical working memory brain networks (Meyer et al., 2012). Here, we review evidence in support of the idea that demanding social cognition may require SWM, that individual differences in neural responses to SWM may explain variance in individual differences in social cognitive ability, and suggest that research on SWM may help address remaining gaps or untested assumptions in social cognition research, as well create novel ways to improve social cognitive function.

## What is Social Working Memory?

Social working memory is working memory for social cognitive information, and will tend to engage during a process referred to as “mentalizing.” Mentalizing is an umbrella term used to describe the ability to think about mental states, traits, beliefs, and intentions (Frith and Frith, 2003). Arguably, complex mentalizing depends on some form of working memory: when considering and attributing mental states to the self and others, people must access, maintain, and manipulate information about the person (self or other) and draw some sort of conclusion about their related mental state. This is similar to the idea that when solving a math problem, people must access and hold representations of the numbers to be manipulated in order to derive an answer (Siegler, 1987, 1988; Geary and Burlingham-Dubree, 1989; Geary and Wiley, 1991; Geary et al., 1993; Ackerman, 1996; Timmermans and Van Lieshshout, 2003; Bjorklund et al., 2004) – and indeed, arithmetic computation is inextricably linked to working memory (e.g., Geary et al., 2004; Wu et al., 2008; Meyer et al., 2010).

Behavioral evidence for SWM comes from studies showing that as social cognitive load increases, mentalizing performance decreases (Kinderman et al., 1998; Rutherford, 2004; Apperly et al., 2007), a behavioral profile consistent with working memory research which suggests that working memory is a limited capacity system (see; Miyake and Shah, 1999 for a review). For example, adults show increased errors on mentalizing tasks as a function of the number of embedded beliefs maintained (i.e., “Bob thinks that John knew that Mary wanted to go to the shop”; Kinderman et al., 1998). Although previously not specified as working memory tasks, such multiple embedding tasks could be conceived of in terms of working memory processes. That is, a correct answer to questions about what Bob thinks requires not only difficult grammatical constructs, but also the active maintenance of belief and desire representations for John and Mary. Likewise, dual-task methodology has shown that performing mentalizing tasks while simultaneously engaging in auditory (McKinnon and Mascovitch, 2007) and verbal working memory (Gilbert et al., 1988) decreases accuracy on various kinds of mentalizing tasks (e.g., theory of mind ToM, trait inference).

**Figure 1:**
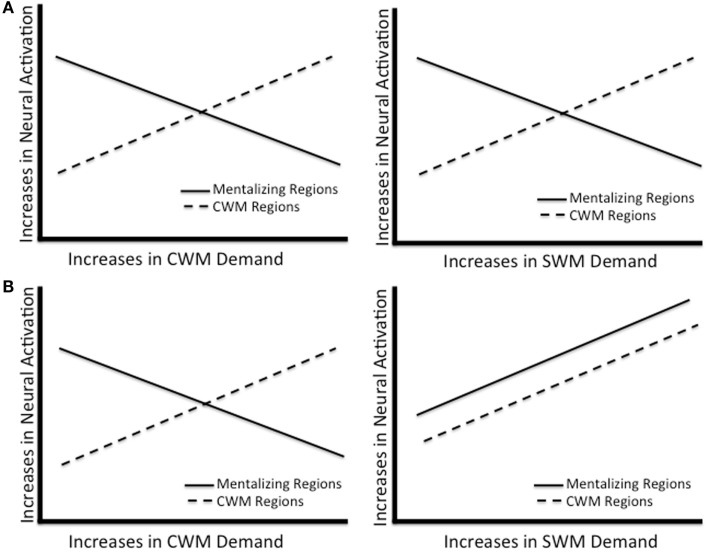
**Models of how the CWM and mentalizing regions respond to working memory demand**. **(A)** Reflects the possibility that mentalizing regions do not support any working memory. **(B)** Reflects the possibility that mentalizing regions do not support non-social working memory, but do support social working memory.

The guiding assumption of these approaches is that there is a limited pool of working memory resources and depleting the pool, either concurrently or prior to mentalizing, reduces the resources available for performing the mentalizing task. In fact, the “cognitive load” method in social cognition (i.e., manipulating CWM demand concurrently or prior to performing a social cognition task and measuring how load effects performance; Gilbert et al., 1988) and the “strength models” of cognitive resources (i.e., that there is one pool of limited resources supporting effortful cognition; Baumeister et al., 1998) seem to depend on this assumption. An alternative hypothesis, however, is that these tasks reflect different patterns of working memory system exhaustion, although both forms correspond with deteriorating mentalizing performance (i.e., different means to the same end). In this scenario, studies that ramp up social cognitive load may exhaust both a specialized SWM system and general CWM system, whereas those enhancing CWM demands may direct attention away from social information processing mechanisms toward CWM mechanisms (see Figure 1B). As will be shown in the subsequent section, brain imaging evidence suggests that this alternative hypothesis may better explain otherwise undetectable mechanistic differences guiding similar behavioral performance in mentalizing across social and non-social load manipulations.

## Mentalizing and Cognitive Working Memory Recruit Distinct Neurocognitive Networks

### Social cognition and the mentalizing system

Mentalizing, or thinking about the psychological characteristics of others, whether it is thinking about their current mental state (beliefs, desires, intentions) or psychological aspects of their personality (traits), reliably recruits functional activation in the so-called “mentalizing network” consisting of medial frontoparietal regions [medial prefrontal cortex (MPFC); precuneus/posterior cingulate cortex (PC/PCC) along with temporoparietal junction (TPJ), temporal poles (TP), and posterior superior temporal sulcus (pSTS; Van Overwalle, 2009; Lieberman, 2010)]. This network is recruited when people draw inferences about the mental states of others either by assessing a person’s state of mind, the emotional reactions they are likely to feel in response to particular events, or the likely behaviors they will engage in based on their intentions and current events (Kampe et al., 2003; German et al., 2004; Walter et al., 2004; Ciaramidaro et al., 2007). This network is also reliably recruited when people think about the psychological traits of other people, such as when they are learning about and judging someone’s personality (Mitchell et al., 2004, 2005; Harris et al., 2005; Heberlein and Saxe, 2005). Consistent with these functional findings, recent findings using structural brain imaging suggest that individual differences in the gray matter structures of the mentalizing system correlates with social cognitive competence and even social network size (Powell et al., 2010; Lewis et al., 2011).

Importantly, the functional MRI studies investigating mentalizing compare brain activation in response to easy social cognitive tasks (e.g., deciding whether adjectives like “charming” could be used to describe people) relative to easy non-social tasks (e.g., deciding whether adjectives like “orange” could describe objects). Prior to our study (Meyer et al., 2012), no research had examined whether and how these regions respond to increases in the amount of social information to be maintained or manipulated during mentalizing. As will be revealed in the subsequent sections, hypothesizing how the mentalizing system may respond to increasing demands in social cognition is not straightforward, and examining this question may reveal interesting insights into the functional dynamics of the mentalizing and CWM networks.

### The neurocognitive network supporting CWM and its anti-correlation with the mentalizing network

Despite the lack of research on SWM, there is a longstanding line of research on CWM, which offers a backdrop for examining whether the mentalizing system may respond similarly to regions supporting CWM. In general, CWM is the ability to maintain and manipulate increasing amounts of information at once. In typical studies of CWM, participants are instructed to maintain or manipulate spatial information (the location of shapes) or verbal information (the order of letters in a string; Smith et al., 1996; D’Esposito et al., 1999) and brain imaging studies using these paradigms consistently report activation in a lateral frontoparietal network consisting primarily of dorsolateral prefrontal cortex (DLPFC), lateral parietal cortex, as well as supplementary motor area (SMA; Goldman-Rakic, 1994; D’Esposito et al., 1999; Rypma et al., 1999; Wager and Smith, 2003). Specifically, activity in these regions increases as the amount of information in CWM increases (Braver et al., 1997; Rypma et al., 1999).

**Figure 2:**
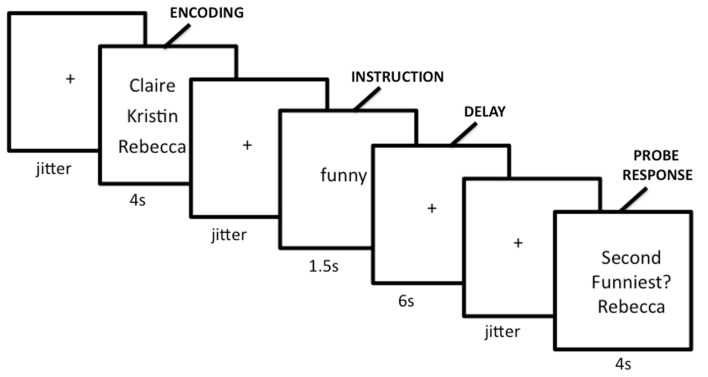
**Social working memory task**.

Importantly though, the neural regions previously implicated in CWM are often thought to be functionally distinct from the brain regions associated with mentalizing (medial frontoparietal cortex, TPJ, TP, and pSTS; e.g., Fox et al., 2005). In fact, brain regions associated with mentalizing seem to interfere with cognitive demands, including working memory (McKiernan et al., 2003; Greicius and Menon, 2004; Li et al., 2007; Anticevic et al., 2010; Metzak et al., 2011). That is, while increased activation in the lateral/SMA network supports cognitive processing, regions in the mentalizing network decrease in activation during cognitive processing. These regions have become known (outside of social neuroscience) as the “default network” (Raichle et al., 2001), so named because they are highly active when participants are passively resting in the scanner (i.e., by default), but show reduced activation, or deactivation, during task performance. Figure 2 shows the anatomical regions associated with the mentalizing network, the default network, and their overlap based on the results from a term-based meta-analysis software, *Neurosynth* (Yarkoni et al., 2011).

A reliable finding is that the default network and lateral frontoparietal network show an inverse pattern of activation both at rest (Shulman et al., 1997; Greicius and Menon, 2004; Fox et al., 2005) and during CWM (Greicius et al., 2003; McKiernan et al., 2003; Metzak et al., 2011). For example, one study manipulated CWM demand with easy, medium, and difficult auditory detection CWM trials. Not only did the CWM network show parametric *increases* in activation, but also regions in the default network showed parametric *decreases* in activation, as a function of CWM trial difficulty (McKiernan et al., 2003). There is also evidence that failure to deactivate regions in the default network during CWM tasks interferes with activating the canonical working memory regions (Greicius and Menon, 2004) and may contribute to poorer cognitive performance (Weissman et al., 2006; Kelly et al., 2008). While these and related findings could suggest that the default network does not support working memory and instead its activation interferes with working memory processes, another possibility is that this network can support working memory when the content is social, and hence anti-correlations with canonical working memory systems is limited to non-social forms of working memory.

Taken together, findings from three literatures offer different pieces of information relevant to understanding SWM. First, social cognitive neuroscience research finds that a specific set of brain regions activate in response to mentalizing (i.e., medial frontoparietal cortex, TPJ, TP, and pSTS; the “mentalizing network”). Second, the CWM literature shows that a different set of brain regions (lateral frontoparietal network and SMA, the “canonical working memory network”) support CWM. Specifically, these regions show a parametric response to CWM demands, which provides a possible pattern to look for when examining which brain regions support SWM. Third, extensive research on the default network shows that there is a network of regions whose functional activation is tightly coupled, most robust at rest, and deactivates during demanding cognition, including CWM. What is fascinating about this literature in the context of SWM is that this default network is anatomically similar to the mentalizing network. Whether and how the mentalizing and CWM networks could support SWM is an intriguing theoretical question, which will be addressed in the subsequent section.

## Mentalizing Under CWM and SWM Load

Most social neuroscience research on mentalizing has used relatively simple mentalizing tasks. When performance measures are reported in fMRI studies of social cognition, they are often near ceiling (Fletcher et al., 1995; Brunet et al., 2000; Walter et al., 2004), implying relative ease. In fact, even 4 year olds achieve high levels of performance on many of the fMRI-based social cognition tasks given to adults (Wimmer and Perner, 1983; Sommer et al., 2007). Therefore, whether the mentalizing network supports complex social cognition, like SWM, remained untested. However, four studies have explored how juggling increased non-social information, or CWM, affects mentalizing system activation during social cognition (den Ouden et al., 2005; Kellermann et al., 2011; Rameson et al., 2012; Spunt and Lieberman, 2012a). For example, requiring participants to maintain a string of numbers while empathizing with others’ emotional states relative to empathizing without maintaining numbers showed reduced activation in several regions of the mentalizing system [e.g., dorsomedial prefrontal cortex (DMPFC), MPFC, VMPFC, PC, pSTS, TP; Rameson et al., 2012]. A similar pattern of results extends to mental state inferences: requiring participants to maintain a complex sequence of digits (e.g., 937–6542) relative to a simple sequence of digits (e.g., 888–8888) while determining characters’ intentions showed reduced activation in mentalizing regions (DMPFC, TP; Spunt and Lieberman, 2012a).

While these studies show that mentalizing regions reduce activation in response to CWM, they cannot speak to how the mentalizing network responds to working memory demands with social cognitive content. Given the fact that this system supports mentalizing, but also deactivates in response to canonical working memory demand (McKiernan et al., 2003; Metzak et al., 2011), it is not entirely clear how the mentalizing network would respond to working memory load in the social domain (i.e., social load). For example, one possibility is that mentalizing regions do not support effortful processing at all. If this were the case, then mentalizing regions may be insensitive, or even reduce activation, in response to demanding social cognition like SWM. This would be consistent with default network characterizations of brain regions associated with mentalizing, whose activation to date has been portrayed as reflecting non-effortful cognitive processing, and even postulated to interfere with effortful cognition (Sonuga-Barke and Castellanos, 2007; Figure 1A). An alternative possibility, however, is that mentalizing regions are specialized to respond to demands in social cognition. Thus, while they *reduce* activation in response to CWM and other non-social forms of effortful cognition, they also *increase* activation in response to SWM and other forms of effortful social cognition (Figure 1B). If this were the case, then mentalizing regions do not support non-effortful cognition *per se* as suggested by the default network literature, but instead support effortful social cognition.

**Figure 3:**
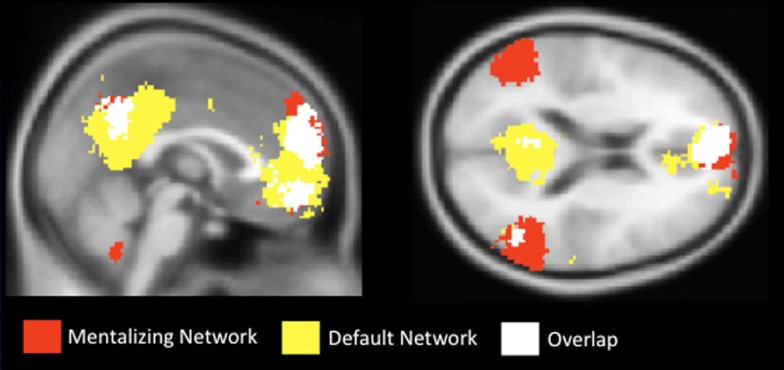
**Results from a term-based search on Neurosynth, which synthesizes fMRI data extracted from published articles**. The mentalizing network, shown in red, was created with the search term “mentalizing.” The default network, shown in yellow, was created with the search term “default.” White areas indicate overlapping areas of activation.

**Figure 4:**
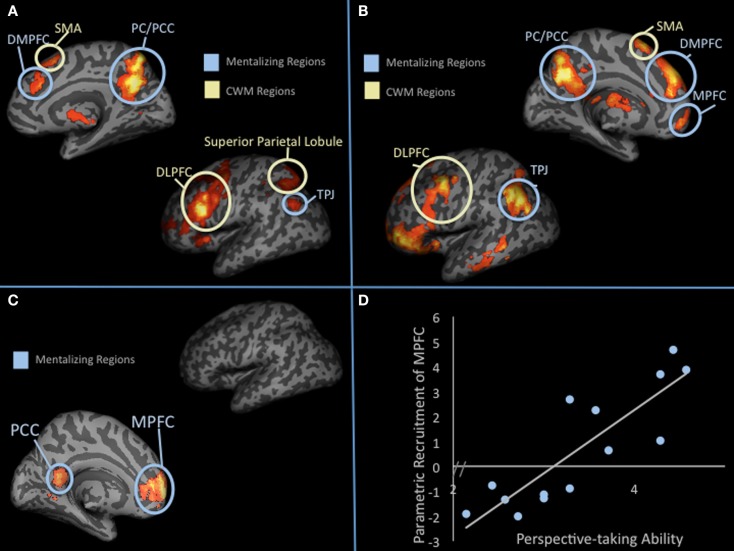
**Parametric increases in response to SWM**. **(A)** shows parametric results (i.e., increases in activation as a function of thinking about 2, 3, or 4 friends) from whole-brain analysis during the delay period. **(B)** Shows parametric results (i.e., increases in activation as a function of thinking about 2, 3, or 4 friends) from whole-brain analysis during the probe-response period. **(C)** Parametric increases in activation as a function of social load during the delay period that correlates with trait perspective-taking scores. **(D)** Graphical display of the correlation shown in **(C)**.

To test these competing hypotheses, we recently examined how the mentalizing regions respond to SWM (Meyer et al., 2012). We developed a delayed-response working memory task that varied working memory load in the social domain on a trial-by-trial basis (Figure 3). During scanning, participants completed trials in which they encoded names of two, three, or four of their friends, mentally ranked their friends along a trait dimension during a delay period, and answered a true/false question about the rankings. Parametric analyses showed increases in the mentalizing system (DMPFC, PC/PCC, TPJ) and canonical working memory system [DLPFC, superior parietal lobule (SPL), SMA] as a function of the number of friends considered along a trait dimension during delay and probe-response periods (Figures 4A,B). Our data are therefore consistent with the hypothesis that the mentalizing regions can support effortful social cognition. Additionally, the CWM network also showed parametric increases in activation suggesting that although these two networks often operate inversely, in the context of SWM they may operate in conjunction. Thus, there may be two separable networks supporting SWM: the mentalizing network, which may be specifically involved in SWM and the canonical working memory network, which may be involved in all known forms of working memory.

These results suggest there might be multiple routes by which secondary tasks inducing CWM or SWM load might impair primary task performance in cognitive or social domains. Given that mentalizing regions commonly involved in social cognition are typically deactivated during effortful cognitive tasks, social load might impair cognitive task performance to the extent that there is negative connectivity between mentalizing and canonical working memory regions. In this hypothesized case, SWM load would not deplete canonical working memory resources, but instead suppress them to the extent to which the two systems are wired to compete. In contrast, non-social cognitive load might deplete canonical working memory controlled processing resources directly. Under many conditions, these two mechanisms might produce similar behavioral outcomes as a function of working memory load, but under others they may not. Connectivity-based suppression versus within system overloading might, for instance, lead to different performance outcomes immediately after the competing demand is eliminated. Future research should examine how the social or cognitive content of secondary tasks differentially lead to resource depletion and whether different neural markers of depletion lead to distinct behavioral responses.

These results are also consistent with a handful of recent studies, which have found that in certain contexts the default network and lateral frontoparietal regions can increase activation in conjunction (see; Spreng, 2012 for review). For example, these networks show positive functional connectivity when participants plan future, personal goals such as “how to find a good job” (Spreng et al., 2010), and co-activation during personal simulations that are goal oriented (Gerlach et al., 2011), creative idea generation (Ellamil et al., 2012), and mind-wandering (Christoff et al., 2009; Christoff, 2012). These studies may link to SWM in a variety of ways. On the one hand, each of these studies involve generating internal, subjective content, and it makes sense to think that SWM does as well: thinking about mental states requires the internal generation of mental state content. On the other hand, each of these past studies may involve social cognition. Even during mind-wandering, we may spontaneously think about our own and others intentions. Another interesting future question to explore is whether one function of the default network is to process social information, and its more natural, externally valid relationship with the lateral frontoparietal network is co-activation to better allow humans to navigate their social world. If this were the case, then past studies showing anti-correlations with the lateral frontoparietal system may be incidental to paradigms that engage demanding cognition for externally invalid stimuli.

While both the mentalizing and CWM networks showed parametric increases in response to SWM load, we found that only parametric increases in the mentalizing network correlated with a standardized measure of perspective-taking ability (Figures 4C,D). Specifically, individuals higher in trait perspective-taking were more likely to show load-dependent parametric increases in MPFC (Brodmann area 10), perhaps suggesting that individuals with greater perspective-taking ability are more able to exert intentional effort in MPFC. Interestingly, this is the only region of the frontal cortex known to be disproportionately larger in humans than other primates after scaling for body size (Semendeferi et al., 2001). In humans, individual differences in MPFC size correlate with social cognitive competence and social network size (Powell et al., 2010; Lewis et al., 2011). To tease apart the causal nature of these kinds of findings, a recent study measured macaque cortex size prior to and after living in groups of 1–7 macaques (Sallet et al., 2011). Rostral prefrontal cortex, a region suggested to be homologous to human BA 10, showed significant increases in gray matter structure as a function of larger group size. Thus, to the extent that the macaque findings parallel human findings, increasing social cognitive demands may cause growth in brain structures like BA 10. Our functional finding and the previous structural findings in humans and macaques dovetail nicely with the social brain hypothesis, which emphasizes that demanding social information processing may have been critical in the expansion of prefrontal cortex size in humans (Dunbar, 1998).

## Future Directions in SWM Research

### Probing the mentalizing system with SWM paradigms

Together, functional and structural findings implicate the mentalizing network in social cognition. Yet the component process each region plays in mentalizing is still an open question, although some speculations have been suggested. In a recent review of mentalizing brain imaging studies, Lieberman (2010) found that across 45 different studies/tasks, DMPFC (BA 8/9) was reported in 91% of studies, whereas TPJ was reported in 59%, TP was reported in 52%, pSTS, and PC/PCC each 39%, and MPFC (BA 10) in 33%, suggesting that DMPFC may play a broad role across different kinds of mentalizing. Interestingly, the second most reported region from the meta-analysis was the TPJ, whose role in mentalizing is still heavily debated. For example, some researchers argue that TPJ activation during mentalizing, over and above attentional demands, reflects social cognitive processing (Young et al., 2010), while others argue that TPJ activation during mentalizing may entirely reflect attention orientation, rather than mentalizing-content-specific computations (Mitchell, 2008). The TPs are highly active when viewing faces and names of familiar people (Sugiura et al., 2006) yet are also associated with semantic knowledge (Schmolck et al., 2002; Bayley and Squire, 2005) and have been proposed to underlie social norm, rule-based or script knowledge guiding mentalizing (e.g., Lambon Ralph et al., 2009; Van Overwalle, 2009). The pSTS, on the other hand, appears to be sensitive to biological motion (e.g., Noguchi et al., 2005), which may help guide inferences about people’s mental states (Baumeister et al., 1998).

Importantly, not only are all of these interpretations relatively speculative, none of them attempt to make sense of these regions’ *interactive roles* in mentalizing. A SWM framework may help unravel the specific component processes these regions may play in mentalizing. For example, one working memory model suggests that a general, or “central executive” system orchestrates the holding of mental representations in mind, while content-specific sub-systems grounded in subdivisions of the parietal cortex code for visuo-spatial and lexical content that is fed forward to the central executive system (Baddeley, 2002). Likewise, demanding social cognitive processing in general, and SWM in particular, may rely on the DMPFC to function as a social-central executive system, whereas TPJ, TP, pSTS, and MPFC may function as SWM sub-systems that feed forward task-specific content. Alternatively, other working memory models suggest that rather than specialized systems dedicated to holding content-specific information in mind, working memory is supported by reactivation of previously stored long-term memories (Anderson, 1983; Cowan, 1995; Ruchkin et al., 2003; Lewis-Peacock and Postle, 2008). It seems possible that mentalizing region activation during SWM may reflect reactivation of previously stored episodic memories, a possibility which could be explored in the future with techniques such multivariate pattern analysis.

In addition to open questions surrounding functional roles of regions within the mentalizing system, the general operating characteristics of this network (i.e., not just *that* regions respond to certain stimuli, but *how* they respond, for example as a linear, quadratic, or cubic function) remains almost entirely unexplored in social neuroscience. Nonetheless, it has become increasingly clear that complex cognitive processes likely emerge from the interactions between and within brain regions that compose networks showing specific functional profiles (Bressler and Menon, 2010). As described previously, there is an overlap between regions of the mentalizing network and one of the core large-scale brain networks typically referred to as the default network (Gusnard et al., 2001; Spreng et al., 2009). Thus, an intriguing future direction for mentalizing research is to move beyond the region-by-region approach and explore how these regions’ functional properties interact as a dynamic network to support mentalizing. SWM paradigms may be particularly useful in this endeavor, as network analyses on subtle manipulations in the mentalizing-content managed in SWM (traits versus beliefs, familiar versus unfamiliar others) may show unique functional relationships across regions within the mentalizing system.

Finally, in everyday life, SWM will often require not only juggling internal mental states, but also external characteristics, such as emotional expression, identity, and action understanding. Studies on working memory for facial identity tend to find increased activation in the fusiform face area (FFA) in addition to activation increases in canonical working memory regions (Druzgal and D’Esposito, 2001, 2003). However, in many of these studies, only facial identity is maintained during a delay. In one study that required the maintenance of facial identity and emotion, the FFA did not show a delay period response. Instead, the amygdala, hippocampus, and lateral orbitofrontal cortex showed sustained increases during the working memory delay period for both identity and emotion trial types (LoPresti et al., 2008). LoPresti et al. (2008) suggested that the FFA may facilitate simple forms of facial working memory, but that it may not be sufficient for maintaining more complex facial information in working memory. An interesting future direction will be to expand on the LoPresti et al. (2008) findings by combining the maintenance of mental states along with emotions and identities to examine how brain regions previously implicated in facial processing interact with the mentalizing system during SWM.

In the context of action identification, a large literature implicates the mirror neuron system, which is neuroanatomically distinct from the mentalizing system, in simulating others’ minds by decoding their behavioral intentions (Aron et al., 1992; Di Pellegriono et al., 1992; Keysers and Gazzola, 2010; Spunt and Lieberman, 2012b). To date, no study has examined the potential working memory properties of the mirror neuron system, although one study has demonstrated that the mirror neuron system may engage more automatically than the mentalizing system (Spunt and Lieberman, 2012a). One interesting possibility is that the mirror neuron and mentalizing system may differ in their working memory properties and future research will be needed to disentangle how we maintain, manipulate, and bind representations of actions and mental states during SWM.

### Informing the debate between mentalizing and executive functions

Mentalizing research has its roots in developmental and comparative psychology that, for several decades, has examined “ToM,” or the *ability* to represent internal mental states (Premack and Woodruff, 1978). A fundamental question in this line of research surrounds how humans are able to understand that people have internal mental states that often times are distinct from our own subjective experience. A longstanding debate in the ToM literature surrounds whether a ToM requires executive functions, or the suite of cognitive abilities including working memory, planning, attention, problem solving, inhibition, and mental flexibility. As it stands, researchers asking this question tend to adhere to one of two sides of a debate. Many suggest that ToM requires executive function ability, including working memory (Hala et al., 2003). Evidence in support of this position comes from (1) developmental findings showing that children on average do not pass the false belief task (a measure of ToM; see; Wimmer and Perner, 1983) until 4 years of age (Gopnik and Astington, 1988), which coincides with the development of working memory (Carlson and Moses, 2001; Tamm et al., 2002); (2) performance on working memory tasks correlates with ToM ability in children (Gordon and Olson, 1998); and (3) adult performance on ToM tasks decreases as a function of task demand (McKiernan et al., 2003; German and Hehman, 2006; Apperly et al., 2007).

Evidence in support of the other side of the debate – that ToM is a specific conceptual knowledge that does not necessitate executive function – (for a discussion, see; Bloom and German, 2000) garners support from brain imaging studies showing that ToM relies on mentalizing regions, rather than brain regions associated with executive function (Baumeister et al., 1998; Lieberman, 2010). In addition, neuropsychological evidence, particularly from research on individuals with Autism Spectrum Disorder (ASD), is consistent with the idea that ToM is distinct from executive functions. Individuals with ASD show deficits in mentalizing, including ToM. However, some evidence suggests that these deficits can persist while executive functions including working memory remain intact (Ozonoff and Strayer, 2001). In addition, children under the age of 4 show improved performance on ToM tasks when cognitive demands are reduced (Lewis and Osborne, 1990; Wellman and Bartsch, 1998; Yazdi et al., 2006), suggesting that the role of executive functions including working memory in ToM may be an artifact of the arbitrary cognitive demands required in false-belief reasoning *per se*, not ToM in particular.

In both sides of the debate, it is assumed that ToM and executive functions are mutually exclusive. An alternative possibility, however, is that mentalizing requires a specific social executive function system, which is distinct from the domain-general executive function system, and is designed to handle increasing amounts of beliefs, traits, and mental states. Consistent with this suggestion, the mentalizing network, which supports ToM, was found to increase linearly as a function of SWM load, as did the CWM system (Meyer et al., 2012). It is possible that parametric increases in the CWM system reflect domain-general working memory demands in the SWM task (i.e., the temporal and spatial ordering of names and/or verbal rehearsal). However, the mentalizing-specific demands (i.e., thinking about the traits of an increasing amount of people) are likely supported by the functionally distinct mentalizing system. In the case of ASD, the domain-general executive functions may be intact, while the domain-specific social executive system may be compromised. Likewise, extant ToM tasks may vary in manipulating social versus cognitive demands and may in turn differentially exhaust one or both executive systems.

### Social cognitive pathologies, social cognitive ability, and interventions

Many psychiatric conditions including schizophrenia, social anxiety, and ASD show dual or differential deficits in social cognition and working memory. Understanding how the mentalizing and CWM networks contribute to SWM may offer important insight into how these systems contribute to various psychological disorders and the kinds of interventions that might benefit them. For example, working memory and ToM are impaired in patients with schizophrenia (Goldman-Rakic, 1994; Pickup and Frith, 2001; Couture et al., 2006). Individuals with social anxiety show working memory deficits, but enhanced working memory for socially salient words (Amir and Bomyea, 2011). Similarly, a hallmark of autism is the impaired ability to relate to and take the perspective of others (Baron-Cohen et al., 1985; Dawson and Fernald, 1987). Interestingly, research on working memory capacity in individuals with ASD is mixed (Bennetto et al., 1996; Russell et al., 1996; Ozonoff and Strayer, 2001; Williams et al., 2006), with some research finding that working memory capacity is relatively intact in high-functioning individuals (Bennetto et al., 1996; Ozonoff and Strayer, 2001). It is possible that social cognitive deficits in these and other disorders may be better characterized with the inclusion of a social cognition task like SWM that varies in difficulty level.

Outside of psychopathology, SWM capacity may also explain variance in healthy individual’s social cognitive abilities and broader “social intelligence.” If, as suggested by Bower (1975), the purpose of working memory “is to build up and maintain an internal model of the immediate environment and what has been happening in our world,” then SWM should be similarly significant. In daily life, much of what one would qualify as “happening” that should require working memory comprises information about people’s psychological characteristics, their mental states, and the relation of these across individuals. On the cognitive side, working memory capacity and lateral frontoparietal activity has been linked to cognitive abilities ranging from math and reading to IQ (Daneman and Carpenter, 1980; Conway et al., 2003; Geary et al., 2004). Similarly, with our SWM paradigm, we identified regions that increased with social load and showed a positive association with self-reported perspective-taking ability (Davis, 1983). Although both lateral and medial frontoparietal networks increased with load in this task, only medial frontoparietal regions showed significant correlations with self-reported perspective-taking ability (see Figures 4B,C). Additionally, medial frontoparietal regions only showed this effect when load-level was taken into account. General responses, collapsing across load-level, showed no correlation with trait perspective-taking ability. Perspective-taking is considered an effortful social cognitive process (Davis et al., 1996; Epley et al., 2004) and greater self-reported perspective-taking is associated with better social functioning (Davis, 1983). This finding is therefore consistent with the suggestion that individual differences in medial frontoparietal activation during SWM may explain variance in real-world social cognitive ability.

Paralleling recent findings with working memory (Klingberg et al., 2005; Jaeggi et al., 2008, 2011), it is also plausible that SWM training could benefit the everyday social cognitive success of individuals with social cognitive deficits and even individuals with normal social cognitive performance. A handful of recent studies, while controversial (for critical reviews of WM training transfer effects see: Melby-Lervåg and Hulme, 2012; Redick et al., 2012; Shipstead et al., 2012), suggest that working memory training not only improves working memory, but these improvements generalize to improved cognitive reasoning and fluid intelligence (Klingberg et al., 2005; Jaeggi et al., 2008, 2011). For example, after completing a working memory intervention, children with attention-deficit hyperactivity disorder (ADHD) showed improvements in working memory capacity, response inhibition, and complex reasoning. In addition, the participants’ parents reported that their children’s ADHD symptoms improved both post-training and after a 3 month follow-up assessment (Klingberg et al., 2005). Similarly, Jaeggi et al. (2008) found that in psychologically healthy adults with normal IQ, working memory training corresponded with improvements in fluid intelligence, or the ability to reason and solve new problems independent of previously acquired knowledge. While preliminary, these findings suggest that working memory ability may be plastic, and that training working memory may help to improve other forms of general cognitive reasoning. By extension, SWM training may be a way to improve both SWM (i.e., how many people can someone think about at once) and other forms of social cognitive reasoning (i.e., perspective-taking) in both atypical and typical populations.

Another interesting hypothesis related to enhancing cognitive ability is the potentially greater efficiency of SWM, relative to non-SWM. Other cognitive operations are facilitated when put in a social context. For example, performance on the Wason card selection task, a measure of conditional reasoning, improves when conditional rules are based on social contracts relative to non-social contingencies (see Cosmides and Tooby, 1992). Similarly, because social cognition may come more readily to individuals, engaging SWM may facilitate recruitment of the lateral frontoparietal working memory network via the mentalizing system, and improve working memory performance.

## Conclusion

Working memory research has focused on the basic building blocks that allow us to handle representations of our immediate environment, but has neglected to incorporate relevant social information that makes up much of our mental processing. Research reviewed here suggests that demanding mentalizing can be conceived as requiring SWM. Interestingly, the picture that is beginning to emerge is that SWM may rely on two functionally distinct neurocognitive networks: The mentalizing network and the canonical working memory network. While the mentalizing network reduces activation under CWM load, it appears to increase activation, alongside the canonical working memory system, while under SWM load. These findings have theoretical implications for the functional properties of the default network and the neural systems that support social cognition, as well as practical implications for future research in social cognitive training.

## Conflict of Interest Statement

The authors declare that the research was conducted in the absence of any commercial or financial relationships that could be construed as a potential conflict of interest.
